# Supplementary material for: LRG1 Associates with Iron Deficiency Anemia Markers in Adolescents
Source: Nutrients. 2023 Jul 11;15(14):3100. doi: 10.3390/nu15143100 (PMC10384480; doi:10.3390/nu15143100)
Supplement: Supplementary file 1 [file nutrients-15-03100-s001.zip › nutrients-2429839-supplementary.pdf]

Supplementary tables

Supplementary Table 1. Hematological parameters differences based on gender.

Characteristic	Female, N = 234[1]	Male, N = 197[1]	p-value[2]
RBC (×1012cells/L)	4.99 (4.73, 5.21)	5.10 (4.86, 5.37)	0.003
WBC (×10^9 cells/L)	6.70 (5.70, 8.30)	6.50 (5.34, 8.10)	0.14
Hb (g/dl)	131 (125, 136)	134 (127, 140)	<0.001
Hct (L/L)	0.403 (0.389, 0.416)	0.408 (0.390, 0.422)	0.089
Gluc (mmol/L)	4.80 (4.50, 5.30)	4.90 (4.40, 5.40)	0.8

[1]Median (IQR)

[2]Wilcoxon rank sum test

Supplementary Table 2. The distribution of anemia subjects *based on gender**.***

**Characteristic**	**Female**, N = 234[1]	**Male**, N = 197[1]	**p-value**[2]
			0.3
Anemic	21 (9.0%)	12 (6.1%)	
Not-anemic	212 (91%)	185 (94%)	

[1]n (%)

[2]Pearson's Chi-squared test

***Supplementary Table 3*. Statistical comparison of LRG across tertiles of CRP, Hb and MCV.**

Characteristic	LRG	p-value[2]
Hb		0.019
74.0-128.0	30 (25, 40)	
128.0-136.0	27 (22, 35)	
136.0-159.0	29 (22, 33)	
CRP		<0.001

Characteristic	LRG	p-value[2]
0-0.28	27 (21, 33)	
0.28-1.54	27 (22, 34)	
1.54-9.89	30 (26, 37)	
MCV (fL)		0.5
56.4-78.6	30 (22, 37)	
78.6-83.2	30 (24, 36)	
83.2-106.5	28 (22, 36)	
[1]Median (IQR)		
[2]Kruskal-Wallis rank sum test		

***Supplementary Table 4*. Comparison of various clinical characteristics based on anemia status.**

Characteristic	Anemic, N = 33[1]	Not-anemic, N = 397[1]	p-value[2]
Gluc (mmol/L)	5.00 (4.70, 5.40)	4.80 (4.40, 5.30)	0.14
Albumin g/liter	40.10 (38.50, 41.80)	41.60 (40.00, 42.83)	0.002
Iron (µg/dL)	9.0 (6.0, 13.0)	13.0 (9.0, 17.0)	0.002
Transferrin saturation (in %)	13 (7, 20)	17 (13, 24)	0.010
Ferritin (µg/L)	13 (6, 33)	21 (15, 31)	0.021
WBC ($\times 10^9$ cells/L)	6.70 (5.70, 7.70)	6.68 (5.50, 8.20)	0.8
RBC (×1012cells/L)	4.88 (4.52, 5.52)	5.02 (4.81, 5.25)	0.6
Hb (g/dl)	113 (107, 117)	133 (128, 139)	<0.001
Hct (L/L)	0.363 (0.347, 0.370)	0.407 (0.394, 0.420)	<0.001
MCV	71 (65, 79)	81 (78, 85)	<0.001
RDW (fL)	15.70 (13.90, 17.20)	13.50 (12.90, 14.20)	<0.001
CRP	0.29 (0.12, 2.54)	0.65 (0.17, 2.10)	0.5
LRG ug/mL	31 (25, 42)	29 (23, 35)	0.07

Characteristic	Anemic, N = 33[1]	Not-anemic, N = 397[1]	p-value[2]
[1]Median (IQR); n (%)			
[2]Wilcoxon rank sum test; Pearson's Chi-squared test; Fisher's exact test			
